# Overview of the diagnostic value of biochemical markers of liver fibrosis (FibroTest, HCV FibroSure) and necrosis (ActiTest) in patients with chronic hepatitis C

**DOI:** 10.1186/1476-5926-3-8

**Published:** 2004-09-23

**Authors:** Thierry Poynard, Françoise Imbert-Bismut, Mona Munteanu, Djamila Messous, Robert P Myers, Dominique Thabut, Vlad Ratziu, Anne Mercadier, Yves Benhamou, Bernard Hainque

**Affiliations:** 1 Groupe Hospitalier Pitié-Salpêtrière, 47-83 Boulevard de l'Hôpital, 75651 Paris Cedex 13, France

## Abstract

**Background:**

Recent studies strongly suggest that due to the limitations and risks of biopsy, as well as the improvement of the diagnostic accuracy of biochemical markers, liver biopsy should no longer be considered mandatory in patients with chronic hepatitis C. In 2001, FibroTest ActiTest (FT-AT), a panel of biochemical markers, was found to have high diagnostic value for fibrosis (FT range 0.00–1.00) and necroinflammatory histological activity (AT range 0.00–1.00). The aim was to summarize the diagnostic value of these tests from the scientific literature; to respond to frequently asked questions by performing original new analyses (including the range of diagnostic values, a comparison with other markers, the impact of genotype and viral load, and the diagnostic value in intermediate levels of injury); and to develop a system of conversion between the biochemical and biopsy estimates of liver injury.

**Results:**

A total of 16 publications were identified. An integrated database was constructed using 1,570 individual data, to which applied analytical recommendations. The control group consisted of 300 prospectively studied blood donors. For the diagnosis of significant fibrosis by the METAVIR scoring system, the areas under the receiver operating characteristics curves (AUROC) ranged from 0.73 to 0.87. For the diagnosis of significant histological activity, the AUROCs ranged from 0.75 to 0.86. At a cut off of 0.31, the FT negative predictive value for excluding significant fibrosis (prevalence 0.31) was 91%. At a cut off of 0.36, the ActiTest negative predictive value for excluding significant necrosis (prevalence 0.41) was 85%. In three studies there was a direct comparison in the same patients of FT *versus *other biochemical markers, including hyaluronic acid, the Forns index, and the APRI index. All the comparisons favored FT (P < 0.05). There were no differences between the AUROCs of FT-AT according to genotype or viral load. The AUROCs of FT-AT for consecutive stages of fibrosis and grades of necrosis were the same for both moderate and extreme stages and grades. A conversion table was constructed between the continuous FT-AT values (0.00 to 1.00) and the expected semi-quantitative fibrosis stages (F0 to F4) and necrosis grades (A0 to A3).

**Conclusions:**

Based on these results, the use of the biochemical markers of liver fibrosis (FibroTest) and necrosis (ActiTest) can be recommended as an alternative to liver biopsy for the assessment of liver injury in patients with chronic hepatitis C. In clinical practice, liver biopsy should be recommended only as a second line test, *i.e.*, in case of high risk of error of biochemical tests.

## Background

One of the major clinical problems is how to best evaluate and manage the increasing numbers of patients infected with the hepatitis C virus (HCV) [1]. Liver biopsy is still recommended in most patients [2,3]. However, numerous studies strongly suggest that due to the limitations [4-6] and risks of biopsy [7], as well as the improvement of the diagnostic accuracy of biochemical markers [8,9], liver biopsy should no longer be considered mandatory.

Among the non-invasive alternatives to liver biopsy [10], several studies have demonstrated the predictive value of two combinations of simple serum biochemical markers in patients infected with HCV: FibroTest (FT; Biopredictive, Paris, France; HCV-Fibrosure, Labcorp, Burlington, USA) for the assessment of fibrosis; and ActiTest (AT; Biopredictive, Paris, France) for the assessment of necroinflammatory activity (necrosis) [8,9,11-21]. Similar results have not been obtained with other diagnostic tests [10-17]. Since September 2002 these tests (FT-AT) have been used in several countries as an alternative to liver biopsy. In a recent systematic review, it was concluded that these panels of tests might have the greatest value in predicting fibrosis or cirrhosis [10]. It was also stated that biochemical and serologic tests were best at predicting no or minimal fibrosis and at predicting advanced fibrosis/cirrhosis, and were poor at predicting intermediate levels of fibrosis [10].

The aim of this study was to summarize the diagnostic value of these tests by an overview of the scientific literature and to respond to the following frequently asked questions by performing original new analyses: 1) what is the range of the FT-AT diagnostic values across the different studies? 2) What are the base evidence comparisons between FT-AT and other published biochemical markers? 3) Are there differences in diagnostic values according to HCV genotype or viral load? 4) Are there differences between the FT-AT diagnostic values according to stages and grades? – In other words, is FT better at predicting no or minimal fibrosis (F0 *vs *F1) or advanced fibrosis/cirrhosis (F3 *vs *F4) than at predicting intermediate levels of fibrosis (F1 *vs *F2)? And 5) what is the conversion between FT-AT results and the corresponding fibrosis stages and necrosis grades?

## Results

### Analysis of the literature

Between February 2001 and March 2004, a total of 16 publications [8,9,11-21,24-26] and 4 abstracts [27-30] without corresponding publications were identified.

### Diagnostic value of FT-AT among published studies

**Figure 1:**
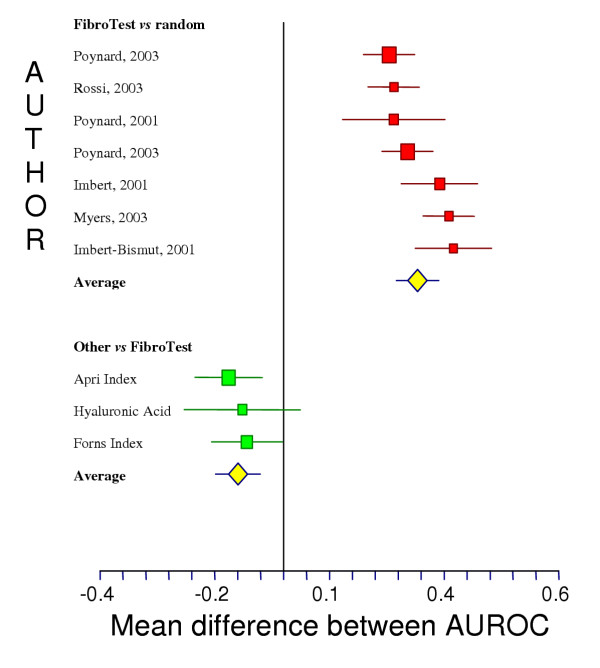
**Meta-analysis of the AUROC observed in published studies of FibroTest diagnostic value. **AUROCs were all significantly higher for FibroTest than the random 0.50 value (upper panel) (P < 0.001). AUROCs of FibroTest were significantly higher then AUROCs of other fibrosis markers (lower panel) (P < 0.05).

For 12 groups of patients detailed in 6 publications [8,11,12,14,19,26], it was possible to assess the prevalence of significant fibrosis and the FT area under receiver operating characteristics curve (AUROC) values, as well as the sensitivity and specificity for the 4 different FT cut offs (Table 1). For the diagnosis of significant fibrosis by the METAVIR scoring system, the AUROC ranged from 0.73 to 0.87, significantly different from random diagnosis in each study (Table 1), in meta-analysis (mean difference in AUROC = 0.39, random effect model Chi-square = 529, P < 0.001) (Figure 1, upper panel), or after pooling data in the integrated database (Table 2). For the cut off of 0.31, the FibroTest negative predictive value for excluding significant fibrosis (prevalence 0.31) was 91% (Table 2).

**Table 1:** Summary of the diagnostic value of FibroTest for the staging of hepatic fibrosis and comparisons with hyaluronic acid, the Forns Index and the APRI Index in patients with chronic hepatitis C, from the published studies.

**First author**	N*	**Methodology**	**Marker**	**Stage/Prevalence**	**AUROC SE**	**Cut off**	**Sensitivity**	**Specificity**
Imbert-Bismut, 2001	189	Prospective Single center First year cohort	FibroTest	F2F3F4 / 0.38	0.84 (0.03)	0.10 0.30 0.60 0.80	0.97 0.79 0.51 0.29	0.24 0.65 0.94 0.95
Imbert-Bismut, 2001	134	Prospective Single center Validation cohort	FibroTest	F2F3F4 / 0.45	0.87 (0.03)	0.10 0.30 0.60 0.80	1.00 0.87 0.70 0.38	0.22 0.59 0.95 0.97
Poynard, 2001	165	Retrospective Randomized trial Multicenter	FibroTest	F3F4 Knodell / 0.32	0.74 (0.03)	0.10 0.30 0.60 0.80	0.96 0.81 0.50 0.13	0.24 0.65 0.92 0.98
Poynard, 2001	165	Retrospective Randomized trial Multicenter	Hyaluronic	F3F4 Knodell / 0.32	0.65 (0.03)	20 40 100	0.81 0.47 0.23	0.39 0.65 0.91
Poynard, 2003	352	Retrospective Randomized trial Multicenter Before treatment	FibroTest	F2F3F4 / 0.39	0.73 (0.03)	0.10 0.30 0.60 0.80	0.97 0.86 0.50 0.20	0.08 0.45 0.79 0.95
Poynard, 2003	352	Retrospective Randomized trial Multicenter After treatment	FibroTest	F2F3F4 / 0.32	0.77 (0.03)	0.10 0.30 0.60 0.80	0.98 0.85 0.46 0.16	0.15 0.39 0.81 0.97
Rossi, 2003	125	Prospective Multicenter Non-validated analyzers	FibroTest	F2F3F4 / 0.38	0.74 (0.05)	0.10 0.30 0.60 0.80	0.92 0.75 0.42 0.22	0.29 0.61 0.94 0.96
Myers, 2003	130	Retrospective Single center HCV-HIV Co-infection	FibroTest	F2F3F4 / 0.45	0.86 (0.04)	0.10 0.30 0.60 0.80	0.98 0.90 0.66 0.34	0.17 0.60 0.92 0.96
Thabut, 2003	249	Retrospective Single center From Imbert-Bismut, 2001	FibroTest	F2F3F4 / 0.38	0.84 (0.02)	0.10 0.30 0.60 0.80	0.98 0.84 0.58 0.29	0.22 0.65 0.93 0.95
Thabut, 2003	249	Retrospective Single center From Imbert-Bismut, 2001	Forns Index	F2F3F4 / 0.38	0.78 (0.03)	1 3 6 8	1.00 1.00 0.55 0.19	0.04 0.26 0.86 0.97
Le Calvez, 2004	323	Retrospective Single center From Imbert-Bismut, 2001	FibroTest	F2F3F4 / 0.41	0.83 (0.02)	0.10 0.30 0.60 0.80	0.97 0.81 0.58 0.33	0.30 0.66 0.93 0.95
Le Calvez, 2004	323	Retrospective Single center From Imbert-Bismut, 2001	APRI Index	F2F3F4 / 0.41	0.74 (0.03)	0.50 1.00 1.50 2.00	0.81 0.54 0.36 0.24	0.56 0.84 0.91 0.95
Callewaert, 2004	82	Prospective	FibroTest	F4 / 0.29	0.89 (0.04)	0.10 0.30 0.60 0.80	1.00 0.92 0.79 0.67	0.33 0.62 0.81 0.92
Callewaert, 2004	82	Prospective	Glyco Cirrho Test	F4 ** / 0.29	0.87 (0.04)	-0.2 0.1 0.4 0.6	1.00 0.79 0.21 0.17	0.12 0.88 0.95 1.00

* Number of patients. ** Compensated

**Table 2:** Integrated database, with predictive values for significant hepatic fibrosis according to METAVIR conversion cut offs. Derived from published studies.

**Integrated database**	**Patient number**	**Marker**	**Stage/Prevalence**	**AUROC (SE)**	**Cut off used for METAVIR stages conversion**	**Sensitivity**	**Specificity**	**Negative predictive value**	**Positive predictive value**
With Blood Donors	1,570	FibroTest	F2F3F4/0.31	0.83 (0.01)	0.21	0.92	0.55	0.94	0.48
					0.27	0.87	0.62	0.92	0.51
					0.31	0.84	0.68	0.91	0.54
					0.48	0.68	0.81	0.85	0.61
					0.58	0.56	0.87	0.82	0.67
					0.72	0.38	0.95	0.77	0.76
					0.74	0.35	0.95	0.76	0.76
					0.75	0.33	0.96	0.76	0.78
Without blood donors	1,270	FibroTest	F2F3F4/0.38	0.78 (0.01)	0.21	0.92	0.41	0.89	0.49
					0.27	0.87	0.48	0.86	0.51
					0.31	0.84	0.55	0.85	0.54
					0.48	0.68	0.73	0.79	0.61
					0.58	0.56	0.83	0.75	0.67
					0.72	0.38	0.95	0.70	0.76
					0.74	0.35	0.93	0.70	0.76
					0.75	0.33	0.94	0.69	0.78

For four groups of patients detailed in two publications [8,11], it was possible to assess the prevalence of significant necrosis and the AT AUROC values, as well as the sensitivity and specificity for 4 different AT cut offs (Table 3). For the diagnosis of significant necrosis by the METAVIR scoring system, the AUROC ranged from 0.75 to 0.86, significantly different from random diagnosis in each study (Table 3), in meta-analysis (mean difference in AUROC = 0.29, random effect model Chi-square = 556, P < 0.001), or after pooling data in the integrated database (Table 4). For the cut off of 0.36, the ActiTest negative predictive value for excluding significant necrosis (prevalence 0.41) was 85% (Table 2).

**Table 3:** Summary of the diagnostic value of ActiTest for the diagnosis of necroinflammatory hepatic activity (AUROC) in patients with chronic hepatitis C, from the published studies.

**First author, Year**	**Patient number**	**Methodology**	**Marker**	**Grade/Prevalence**	**AUROC (SE)**	**Cut off**	**Sensitivity**	**Specificity**
Imbert-Bismut, 2001	189	Prospective Single center	ActiTest	A2A3 / 0.33	0.79 (0.03)	0.10 0.30 0.60 0.80	0.99 0.91 0.70 0.49	0.07 0.42 0.75 0.88
Imbert-Bismut, 2001	134	Prospective Single center Validation cohort	ActiTest	A2A3 / 0.28	0.75 (0.03)	0.10 0.30 0.60 0.80	1.00 0.94 0.67 0.42	0.07 0.33 0.65 0.87
Poynard, 2003	352	Retrospective Randomized trial Multicenter Before treatment	ActiTest	A2A3 / 0.83	0.75 (0.03)	0.10 0.30 0.60 0.80	1.00 0.90 0.49 0.20	0.00 0.38 0.87 0.99
Poynard, 2003	352	Retrospective Randomized trial Multicenter After treatment	ActiTest	A2A3 / 0.39	0.86 (0.02)	0.10 0.30 0.60 0.80	0.91 0.75 0.38 0.14	0.59 0.83 0.98 0.996

**Table 4:** Integrated database, with predictive values for the diagnosis of significant necroinflammatory hepatic activity according to METAVIR conversion cut offs. Derived from published studies.

**Integrated database**	**Patient number**	**Marker**	**Grade/Prevalence**	**AUROC (SE)**	**Cut off used for METAVIR stages conversion**	**Sensitivity**	**Specificity**	**Negative predictive value**	**Positive predictive value**
With Blood Donors	1,570	ActiTest	A2A3/0.41	0.85 (0.01)	0.17	0.95	0.55	0.94	0.60
					0.29	0.87	0.69	0.88	0.66
					0.36	0.81	0.74	0.85	0.69
					0.52	0.62	0.86	0.76	0.75
					0.60	0.51	0.90	0.72	0.77
					0.61	0.50	0.90	0.72	0.78
					0.62	0.49	0.91	0.72	0.78
Without blood donors	1,270	ActiTest	A2A3/0.51	0.78 (0.01)	0.17	0.95	0.40	0.89	0.62
					0.29	0.87	0.55	0.80	0.67
					0.36	0.81	0.63	0.76	0.69
					0.52	0.62	0.79	0.67	0.75
					0.60	0.51	0.85	0.63	0.77
					0.61	0.50	0.85	0.62	0.78
					0.62	0.49	0.86	0.62	0.78

### Comparison of FT-AT diagnostic values with other biochemical markers

In four studies there was a direct comparison in the same patients of FT *versus *other biochemical markers, including hyaluronic acid [12], the Forns index [16], the APRI index [17] and the GlycoCirrhoTest [26]. All the comparisons were in favor of FT (Table 1) (Figure 1, lower panel), except for the GlycoCirrhoTest, which has a similar AUROC (0.87 *vs *0.89 for FT) [26].

### Integrated database

A total of 1,570 subjects were included in the integrated database. Of these, 1,270 were patients with chronic hepatitis C who tested PCR positive before treatment and who had had a liver biopsy and METAVIR staging and grading performed. Of these patients, 453 were from our center [11,14], including 130 patients coinfected with HCV and HIV [14]. Eight hundred and seventy (870) patients were from a multicentre study with a total of 398 patients assessed at inclusion and 419 at the end of follow-up six months after treatment; 352 being investigated twice. Three hundred (300) healthy blood donors were also included [20].

### Diagnostic value of FT-AT according to HCV genotype and viral load

**Figure 2:**
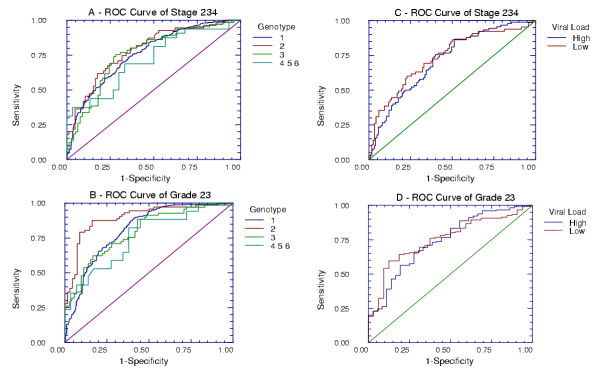
**Diagnostic values of FibroTest according to genotype and viral load. **Graph A: AUROCs of FibroTest for the diagnosis of significant fibrosis, according to HCV genotypes. There were no significant differences: Genotype 1, n = 684, AUROC = 0.76, 95% Confidence Interval (95CI) = 0.72–0.79; genotype 2, n = 140, AUROC = 0.79, 95CI = 0.70–0.85; genotype 3, n = 143 AUROC = 0.76, 95CI = 0.67–0.83; other genotype, n = 46, AUROC = 0.72, 95CI = 0.52–0.85. Graph B: AUROCs of ActiTest for the diagnosis of significant necrosis, according to HCV genotypes. There were no significant differences: Genotype 1, n = 684, AUROC = 0.81, 95% Confidence Interval (95CI) = 0.77–0.84; genotype 2, n = 140, AUROC = 0.90, 95CI = 0.83–0.94; genotype 3, n = 143, AUROC = 0.79, 95CI = 0.71–0.85; other genotype, n = 46, AUROC = 0.76, 95CI = 0.57–0.87. Graph C: AUROCs of FibroTest for the diagnosis of significant fibrosis, according to serum HCV viral load. There were no significant differences: High viral load, n = 215, AUROC = 0.71, 95% Confidence Interval (95CI) = 0.64–0.78; Low viral load, n = 183, AUROC = 0.73, 95CI = 0.65–0.80. Graph D: AUROCs of ActiTest for the diagnosis of significant necrosis, according to serum HCV viral load. There were no significant differences: High viral load, n = 215, AUROC = 0.74, 95% Confidence Interval (95CI) = 0.64–0.82; Low viral load, n = 183, AUROC = 0.75, 95CI = 0.65–0.82.

There was no difference between the AUROC of FT-AT for the diagnosis of significant fibrosis (F2F3F4) (Figure 2A) and significant necrosis (A2A3) (Figure 2B) between 4 classes of genotype (1, 2, 3 and the rarer genotypes 4, 5, 6 grouped together). There was also no difference between the AUROC of FT-AT of patients with high or low viral loads for the diagnosis of significant fibrosis (Figure 2C) or significant necrosis (Figure 2D).

### Diagnostic value of FT according to the independency of authors

Among the 13 published studies of FT (detailed in Table 1), 9 studies estimated FT and 4 studies compared FT to other non-invasive tests. Among the 9 studies estimating FT, 5 were performed by the same single center (non-independent center), two were performed in totally independent centers, and two were performed in multiple centers, including the non-independent center. The AUROCs for the diagnosis of F2F3F4 *versus *random AUROCs at 0.50, were all significant and similar between these 3 groups in a meta-analysis: mean difference in AUROC = 0.29 (random effect model Chi-square = 549, P < 0.001), including 0.24 for independent, 0.25 for mixed and 0.36 for dependent studies. In the Callewaert et al. [26] study the AUROC of FT for the diagnosis of F4 was 0.89.

### Diagnostic value of FT-AT according to stage and grade

The AUROCs between different stage combinations are given in Table 5. Between two contiguous stages (one stage difference), the AUROCs were not significantly different and ranged from 0.63 to 0.71. Between patients with a two-stage difference, the AUROCs were not significantly different and ranged from 0.75 to 0.86. Between patients with a three-stage difference, the AUROCs were not significantly different and ranged from 0.87 to 0.95. Between patients with a four- or five-stage difference (blood donors *versus *F3 or F4, and F0 *versus *F4), the AUROCs were not significantly different and ranged from 0.95 to 0.99.

**Table 5:** Summary of the diagnostic value of FibroTest for the diagnosis of all stage combinations of hepatic fibrosis, according to the AUROCs.

	**F0**	**F1**	**F2**	**F3**	**F4**	**BD F0**	**F0F1**	**F1F2**	**F2F3F4**	**F3F4**
Blood Donor (BD) n = 300	**0.71**	*0.86*	***0.95***	0.99	0.99	-	0.84	0.88	0.97	0.99
F0 n = 95	-									
F1 n = 688	**0.66**	-								
F2 n = 253	*0.82*	**0.69**	-							
F3 n = 111	***0.92***	*0.80*	**0.63**	-						
F4 n = 123	0.95	***0.87***	*0.75*	**0.65**	-					
BD F0	0.71	0.81	0.92	0.98	0.98	-				
F0F1	-	-	0.71	0.82	0.88	-	-	-		
F1F2	0.71	-	0.69	0.81	0.82	0.84	-	-	-	
F2F3	0.85	0.76	-	-	0.72	0.92	0.80	-	-	
F3F4	0.94	0.81	0.81	-	-	0.98	0.89	0.80	-	
F2F3F4	0.83	0.78	-	-	-	0.95	0.78	-	-	-
BD F0F1	-	-	0.77	0.87	0.91	-	-	-	0.83	0.89

The AUROC between all different stage combinations are given. Between two contiguous stages (one- stage difference), the AUROCs are given in bold. Between patients with a two-stages difference, the AUROCs are given in italics. Between patients with a three-stages difference, the AUROCs are given in bold and italics. Between patients with a four- or five-stages difference (blood donors versus F3 or F4, and F0 versus F4), the AUROCs are underlined. Significant differences were observed between AUROCs when there was a two-stage or more difference.

The AUROCs between different grade combinations are given in Table 6. Between two contiguous grades (one grade difference), the AUROCs were not significantly different and ranged from 0.60 to 0.70. Between patients with a two-grade difference, the AUROCs were not significantly different and ranged from 0.75 to 0.86. Between patients with a three-grade difference, the AUROCs were not significantly different and ranged from 0.87 to 0.95. Between patients with a four-grade difference (blood donors *versus *F3 and F0 *versus *F4), the AUROCs were not significantly different and ranged from 0.95 to 0.99.

**Table 6:** Summary of the diagnostic value of ActiTest for the differential diagnosis of all grades of necroinflammatory hepatic activity, according to the AUROCs.

	**A0**	**A1**	**A2**	**A3**	**BD A0**	**A0A1**	**A1A2**	**A2A3**
Blood Donor BD n = 300	**0.67**	*0.84*	0.96	**0.99**	-	0.79	0.89	0.97
A0 n = 185	-							
A1 n = 443	**0.69**	-						
A2 n = 370	*0.87*	**0.70**	-					
A3 n = 272	***0.93***	*0.79*	**0.60**	-				
A0A1	-	-	0.70	0.83	-			
A1A2	0.77	-	-	0.70	0.85	-		
A2A3	0.89	0.74	-	-	0.94	0.78	-	
A0A1A2	-	-	-	0.75	-	-	-	-
BD A0A1	-	-	0.82	0.88	-	-	-	0.84

The AUROCs between all different grade combinations are given. Between two contiguous grades (one-grade difference), the AUROCs are given in bold. Between patients with a two-grades difference, the AUROCs are given in italics. Between patients with a three-grades difference, the AUROCs are given in bold and italics. Between patients with a four- or five-grades difference (blood donors versus F3 or F4, and F0 versus F4), the AUROCs are underlined. Significant differences were observed between AUROCs when there was a two-grade or more difference.

### Conversion between FT-AT results and the corresponding fibrosis stage and grade

**Figure 3:**
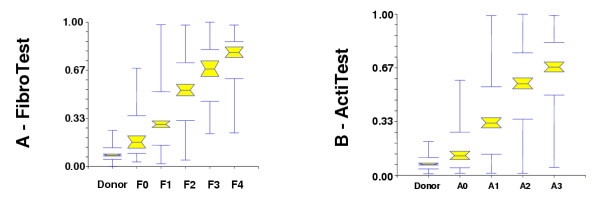
**Conversion between FibroTest and fibrosis stages, and between ActiTest and necroinflammatory activity grades – Graphs. **Graph A: FibroTest values according to status, from blood donors to patients with cirrhosis (n = 1570). Graph B: ActiTest values according to status, from blood donors to patients with severe necrosis (n = 1570). F0 = no fibrosis, F1 = portal fibrosis, F2 = some septa, F3 = many septa, F4 = cirrhosis, A0 = no necroinflammatory activity, A1 = minimal activity, A2 = moderate activity, A3 = severe activity. (Consensus conferences recommend treatment in patients with either F2 stage or A2 grade.) Notched box plots showing the relationship between FibroTest and the stage of fibrosis (A) and between ActiTest and the grade of activity (B). The horizontal line inside each box represents the median, and the width of each box the median ± 1.57 interquartile range/√n (to assess the 95% level of significance between group medians). Failure of the shaded boxes to overlap signifies statistical significance (P < 0.05). The horizontal lines above and below each box encompass the interquartile range (from 25^th ^to 75^th ^percentile), and the vertical lines from the ends of the box encompass the adjacent values (upper: 75^th ^percentile plus 1.5 times interquartile range, lower 25^th ^percentile minus 1.5 times interquartile range).

**Figure 4:**
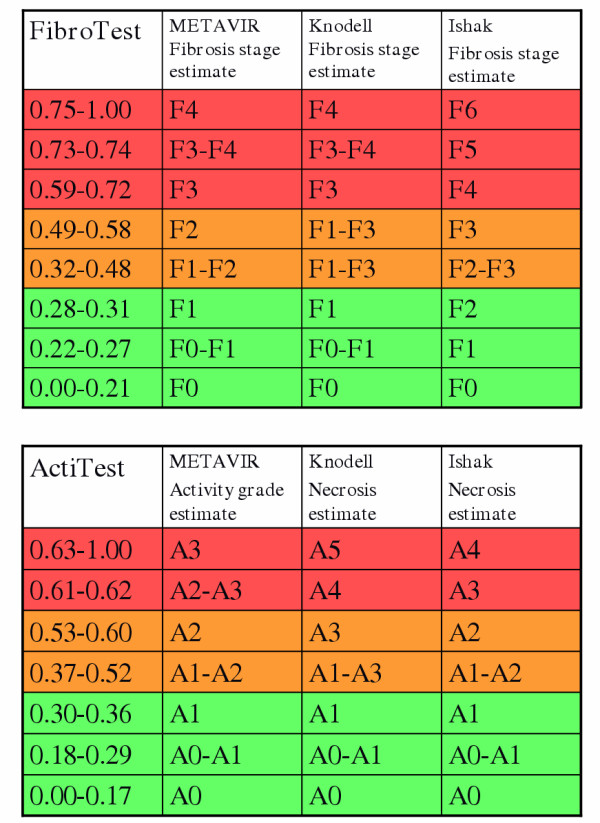
**Conversion between FibroTest and fibrosis stages, and between ActiTest and necroinflammatory activity grades – Panels. **Conversion between FibroTest and fibrosis stages using METAVIR, Knodell and Ishak fibrosis scoring systems (upper panel). Conversion between ActiTest and activity grades using METAVIR, Knodell and Ishak necroinflammatory activity scoring systems (lower panel).

FT-AT is a continuous linear biochemical assessment of fibrosis stage and necroinflammatory activity grade. It provides a numerical quantitative estimate of liver fibrosis ranging from 0.00 to 1.00, corresponding to the well-established METAVIR scoring system of stages F0 to F4 and of grades A0 to A3. Among the 300 controls, the median FT value (± SE) was 0.08 ± 0.004 (95^th ^percentile, 0.23) and the median AT value was 0.07 ± 0.004 (95^th ^percentile, 0.26). Among the 1,270 HCV-infected patients, the FT conversion was 0.000 – 0.2100 for F0; 0.2101 – 0.2700 for F0–F1; 0.2701 – 0.3100 for F1; 0.3101 – 0.4800 for F1–F2; 0.4801 – 0.5800 for F2; 0.5801 – 0.7200 for F3; 0.7201 – 0.7400 for F3–F4; and 0.7401 – 1.00 for F4. (Figure 3A). The AT conversion was 0.00 – 0.1700 for A0; 0.1701 – 0.2900 for A0–A1; 0.2901 – 0.3600 for A1; 0.3601 – 0.5200 for A1–A2; 0.5201 – 0.6000 for A2; 0.6001 – 0.6200 for A2–A3; and 0.6201 – 1.00 for A3 (Figure 3B). The conversions are summarized in Figure 4.

## Discussion

Based on the limitations of liver biopsy and the present overview of the diagnostic value of FT-AT, it seems that these non-invasive markers should be used as a first line assessment of liver injury in patients with chronic hepatitis C.

Liver biopsy has three major limitations, which are the risk of adverse events [2,3,7], sampling error [4-6], and inter- and intra- pathologist variability [23]. An overview of published studies summarizes the risks of liver biopsy as pain (around 30%), severe adverse events (3/1,000) and death (3/10,000) [2,3,7]. Sampling variation is the major cause of variability [4-6]. In a study of patients with chronic hepatitis C that included only good quality biopsies, 30 of 124 patients (24.2%) had a difference of at least one grade, and 41 of 124 patients (33.1%) had a difference of at least one stage between the right and left lobes [4]. In 18 patients (14.5%), an interpretation of cirrhosis was made in one lobe, whereas stage 3 fibrosis was made in the other [4]. Recently, Bedossa et al. [6] observed very high coefficients of variation (55%) and high discordance rates (35%) for fibrosis staging in biopsies measuring 15 mm in length. The variability significantly improved in biopsies measuring 25 mm in length but was still very high with a 45% coefficient of variation and 25% discordance rate; the minimal variability was reached for biopsies, which were 40 mm in length [6].

Liver biopsy has also potential advantages. Biopsy could be of diagnostic value for other unrecognized liver disease. These events are probably rare in practice, as we observed no such a case in a prospective study of 537 consecutive patients with chronic hepatitis C [9]. For FT-AT it must be realized that the same predictive values were observed for patients coinfected with HIV [14], and in patients with other causes of liver fibrosis such as chronic hepatitis B [31], alcoholic liver disease [27] or non-alcoholic steato-hepatitis [27].

It is possible that biochemical markers such as those described here may provide a more accurate (quantitative and reproducible) picture of fibrogenic and necrotic events occurring within the liver than hepatic biopsy. The greater accuracies of FT-AT, when assessed with biopsy specimens greater than 15 mm *versus *smaller biopsies, suggest that some discordance between FT-AT and histology were due to biopsy specimen sampling error [8]. Several case reports have observed false negatives of liver biopsy *versus *biochemical markers [8,9,11]. The error was attributable to biopsy because there were overt clinical signs of cirrhosis such as esophageal varices, low platelet counts or a dysmorphic liver on ultrasound. In a recent prospective study we estimated that 18% of discordances between FT-AT and histology were attributable to biopsy failure (mostly due to small length) and 2% to FT-AT failure [9].

The present work allowed frequently asked questions to be answered, the first being whether the diagnostic values of FT-AT had been confirmed in all studies performed to date. A major strength of the studies pertaining to FT-AT is that they were carried out on a large number of patients with chronic hepatitis C, and the results were reproducible in different populations, including patients coinfected with HIV. There was a small variability in the AUROCs, both for the diagnosis of significant fibrosis (0.73 to 0.87) and significant necrosis (0.75 to 0.86).

A weakness of this study was that the same group, which developed these tests, performed most of the published studies. However the independent published studies found the same significant diagnostic values than non-independent or multicentre studies. Several recent independent studies confirmed the predictive value of FT-AT [26,30].

The second question concerned the comparison of FT-AT to other tests. In their recent review, Gebo et al. [10] concluded that panels of markers might have the greatest value in predicting the absence or no more than minimal fibrosis on biopsy, and in predicting the presence of cirrhosis on biopsy (Evidence Grade B). They pointed out that five studies [11,32-35] used large panels of markers and achieved the greatest predictive values. Among these 5 studies were the first FT-AT study [11] and another study developed by the same group (combining age and platelets) [34]. A recent study compared FT-AT to the age and platelets index in the same patients and found that FT-AT was significantly better [15]. Three studies directly compared FT-AT, to hyaluronic acid [12], the Forns index [16] and the Wai index [17] in the same patients. FT-AT had higher diagnostic values (the AUROC was significantly higher). FT was in particular more sensitive for discriminating between F1 and F2, and more linearly correlated to stages when compared to those 3 other markers [12,16,17]. An additional weakness of the Forns index is the inclusion of cholesterol, which varies greatly in patients with genotype 3 [16]. The limitations of these three comparisons [12,16,17] are that they were retrospective and were performed by the same group. These comparisons, however, had no evident sources of bias. The comparison with the Forns Index [16] included all patients of the Imbert-Bismut *et al. *study (n = 323) [11], as the parameters belong to the routine biochemical tests. The comparison with the APRI index included 249/323 patients (77%) without any difference between included or non-included patients when all characteristics were compared [17]. The comparison with hyaluronic acid [12] included a total of 165 out of the 244 (68%) randomized patients pre-included. The 165 included patients did not differ from the 79 non-included patients according to the main characteristics. Among the 165 patients, the fibrosis index was assessed in 461 samples and hyaluronic acid in 457 samples [12].

Recently, a study using profiles of serum protein N-glycans found that a profile has a similar AUROC than FT for the diagnosis of compensated cirrhosis. When combined with FT this marker had 100% specificity and 75% sensitivity for the diagnosis of compensated cirrhosis, which is not significantly different from the 92% specificity and 67% sensitivity of the FT [26]. This study was independent and prospectively designed for taking FT as the comparison test. Only 24 patients with cirrhosis were included and no details were given concerning the causes of discordance between biopsy and biochemical markers.

However FT-AT is the only panel of markers identified by an independent overview [9], which has been compared in the same patients with most of the other proposed markers. No studies were found that compared FT-AT with a panel of extra-cellular matrix markers [31]. Compared to other panels, FT-AT also allowed an estimation to be made not only of the fibrosis stage but also the necroinflammatory (histological) activity.

The present analysis of the integrated database demonstrated that the diagnostic value of FT-AT did not depend on HCV genotype or viral load. However, because of the small number of patients included, studies in genotype 4, 5 and 6 would be useful.

The present analysis also answered another frequently asked question concerning the predictive values for the intermediate stages of fibrosis. Contrary to the initial hypothesis, the diagnostic values of FT-AT for consecutive stages of fibrosis and grades of necroinflammatory activity were the same for both moderate and extreme stages and grades. Our interpretation is that the same overlap exists between all stages, which is mainly related to the sampling error of the biopsy. It is very reassuring that the medians of FT-AT are linearly associated with stages and grades (Figures 3A,3B). The linearity of this association became even more evident as a larger number of patients were included (data not shown).

Finally, the integrated database allowed a simple conversion system to be proposed to clinicians between liver injury as estimated by the FT-AT and that as estimated by liver biopsy (Figure 4). One conventional way to express the diagnostic values of FT-AT was summarized using the cutoffs of the distribution by stages and grades (Tables 2 and 4). The negative predictive value of FT for excluding significant fibrosis was excellent for the 0.31 cutoff (91%), as was the negative predictive value for excluding significant activity at the 0.36 cutoff of AT (85% negative predictive value). The positive predictive value of the 0.72 cutoff of FT for significant fibrosis was also high at 76%. This, however, may appear lower than the negative predictive value. There is a technical explanation owing to the prevalence of significant fibrosis, which was only 0.31 in this population. According to the excellent specificity (above 0.95), the positive predictive value increased rapidly in populations with more fibrosis (data not shown). We recently observed that the main reason for this was probably because most of the so-called false positives of the FT were in fact false negatives due to the small sampling size of liver biopsies [5,9]. The same comments can be made concerning the positive predictive value of AT for significant necrosis with 77% at the 0.60 cutoff. Again, it is probable that a large proportion of so-called false positives of AT were in fact false negatives due to liver biopsies which were too small. The ideal study would be one using biopsies measuring 40 mm in length, as two samples of 20 mm each during laparoscopy. Only this very high quality biopsy can be considered as a true gold standard. Obviously this type of biopsy cannot be performed routinely as first line, but it could be recommended for clinical research.

## Conclusions

Based on these results, the use of the biochemical markers of liver fibrosis (FibroTest) and necrosis (ActiTest) can be recommended as an alternative to liver biopsy for the first line assessment of liver injury in patients with chronic hepatitis C. In clinical practice, liver biopsy should be recommended only as a second line test, *i.e.*, in case of high risk of error of biochemical tests or in transplanted patients. For clinical research, only very high quality liver biopsy (as two samples of 20 mm each) can be considered as a gold standard for validation of new alternatives.

## Methods

### Analysis of the literature

We did a search for all publications and communications between February 2001 and March 2004 with the key words "FibroTest" and "ActiTest" in Medline and in the abstract books of hepatology, gastroenterology, internal medicine and infectious diseases annual meetings. Only publications or abstracts concerning FT-AT in chronic hepatitis C were included.

### Diagnostic value of FT-AT among published studies

For each study we assessed the diagnostic value for the diagnosis of significant fibrosis (bridging fibrosis or stages F2, F3, F4 according to the METAVIR scoring system) and significant necroinflammatory activity (moderate or severe necrosis, grades A2 or A3 according to the METAVIR scoring system) by the area under the receiver operating characteristics curve (AUROC).

For several databases it was possible to re-analyze the individual data and we looked at the sensitivity and specificity according to different thresholds (0.10, 0.30, 0.60 and 0.80). When FT-AT was compared to other biochemical tests, we also assessed the corresponding sensitivity and specificity according to several thresholds.

### Comparison of FT-AT diagnostic values with other biochemical markers

We selected studies using direct comparisons of diagnostic values in the same patients. The AUROCs were compared for the diagnosis of significant fibrosis (F2F3F4) and significant necrosis (A2A3).

### Integrated database

Patients were included in an integrated database if they belonged to a published population of patients with chronic hepatitis C. Liver biopsy was scored using the METAVIR scoring system and FT-AT was assessed using the recommended pre-analytical and analytical procedures [18,20]. A published population of 300 prospectively analyzed blood donors was included as a control group [20].

### Diagnostic value of FT-AT according to HCV genotype and viral load

Using the integrated database, we compared the AUROCs of FT-AT for the diagnosis of significant fibrosis (F2F3F4) and significant activity (A2A3) between 4 classes of genotype (1, 2, 3 and the rarer genotypes 4, 5, 6 grouped together). For viral load, only those assessed in the same laboratory were included in the comparison between AUROCs, and the median was used to define low and high viral loads (3,800,000 copies/ml) [8].

### Diagnostic value of FT-AT according to stage and grade

Using the integrated database, we compared the diagnostic values according to different stages or grades. We compared the AUROCs for all possible combinations of stages and grades, including combinations with blood donors. This allowed, for example, a comparison to be made of the diagnostic value of FT for discriminating between F1 and F2 after excluding all other stages of the database.

### Liver biopsies

In the integrated database, liver biopsies were processed using standard techniques. A pathologist who was unaware of the biochemical markers evaluated fibrosis stage and necrosis grade according to the METAVIR scoring system [22,23].

Fibrosis was staged on a scale of 0 to 4: F0 = no fibrosis, F1 = portal fibrosis without septa, F2 = few septa, F3 = numerous septa without cirrhosis, F4 = cirrhosis. The grading of activity by the METAVIR system (based on the intensity of necroinflammatory activity, mainly on necrosis) was scored as follows: A0 = no necroinflammatory activity, A1 = mild activity, A2 = moderate activity, A3 = severe activity [22,23].

### Biochemical markers

We used the previously validated FT-AT [8,9,11-21]. FT-AT is a non-invasive blood test that combines the quantitative results of six serum biochemical markers [alpha2-macroglobulin, haptoglobin, gamma glutamyl transpeptidase (GGT), total bilirubin, apolipoprotein A1 and alanine aminotransferase (ALT)] with the patient's age and gender in a patented artificial intelligence algorithm (USPTO 6,631,330) to generate a measure of fibrosis stage and necroinflammatory grade in the liver.

### Statistical analysis

Corresponding stages and grades were calculated from median scores and 95% confidence intervals were observed in 1,270 patients and 300 healthy blood donors. The AUROC was used as a measure of discrimination, estimated using the empirical (non-parametric) method by DeLong et al. [36], and were compared using the paired method by Zhou et al. [36]. All analyses are performed on the NCSS software (Kaysville, Utah) [36].

## Authors' contributions

TP and MM conceived the study, performed the statistical analysis, and wrote the manuscript. FIM, BH and DM carried out biochemical analyses. RP, DT, VR, and YB participated in the coordination of the study, and drafted the manuscript. AM participated in the design and coordination of assays in the control group. All authors read and approved the final manuscript.

## References

[B1] Afdhal NH (2003). Diagnosing fibrosis in hepatitis C: is the pendulum swinging from biopsy to blood tests?. Hepatology.

[B2] Dienstag J (2002). The role of liver biopsy in chronic hepatitis C. Hepatology.

[B3] Bravo AA, Sheth SG, Chopra S (2001). Liver biopsy. N Engl J Med.

[B4] Regev A, Berho M, Jeffers LJ, Milikowski C, Molina EG, Pyrsopoulos NT, Feng ZZ, Reddy KR, Schiff ER (2002). Sampling error and intraobserver variation in liver biopsy in patients with chronic HCV infection. Am J Gastroenterol.

[B5] Colloredo G, Guido M, Sonzogni A, Leandro G (2003). Impact of liver biopsy size on histological evaluation of chronic viral hepatitis: the smaller the sample, the milder the disease. J Hepatol.

[B6] Bedossa P, Dargère D, Paradis V (2003). Sampling variability of liver fibrosis in chronic hepatitis C. Hepatology.

[B7] Poynard T, Ratziu V, Bedossa P (2000). Appropriateness of liver biopsy. Can J Gastroenterol.

[B8] Poynard T, McHutchison J, Manns M, Myers RP, Albrecht J (2003). Biochemical surrogate markers of liver fibrosis and activity in a randomized trial of peginterferon alfa-2b and ribavirin. Hepatology.

[B9] Poynard T, Munteanu M, Imbert-Bismut F, Charlotte F, Messous D, Dominique Thabut D, Thibaut V, Benhamou Y, Ratziu V (2004). Prospective analysis of discordant results between biochemical markers and biopsy in patients with chronic hepatitis C. Clin Chem.

[B10] Gebo KA, Herlong HF, Torbenson MS, Jenckes MW, Chander G, Ghanem KG, El-Kamary SS, Sulkowski M, Bass EB (2002). Role of liver biopsy in management of chronic hepatitis C: A systematic review. Hepatology.

[B11] Imbert-Bismut F, Ratziu V, Laurence Pieroni L, Charlotte F, Benhamou Y, Poynard T, MULTIVIRC Group (2001). Biochemical markers of liver fibrosis in patients with hepatitis C virus infection: a prospective study. Lancet.

[B12] Poynard T, Imbert-Bismut F, Ratziu V, Chevret S, Jardel C, Moussalli J, Messous D, Degos F (2002). Biochemical markers of liver fibrosis in patients infected by Hepatitis C Virus: Longitudinal validation in a randomized trial. J Viral Hepatitis.

[B13] Myers RP, Ratziu V, Imbert-Bismut F, Charlotte F, Poynard T (2002). Biochemical markers of liver fibrosis: a comparison with historical features in patients with chronic hepatitis C. Am J Gastroenterol.

[B14] Myers RP, Benhamou Y, Imbert-Bismut F, Thibault V, Bochet M, Charlotte F, Ratziu V, Bricaire F, Katlama C, Poynard T (2003). Serum biochemical markers accurately predict liver fibrosis in HIV and hepatitis C virus-coinfected patients. AIDS.

[B15] Myers RP, de Torres M, Imbert-Bismut F, Ratziu V, Charlotte F, Poynard T (2003). Biochemical markers of fibrosis in patients with chronic hepatitis C: a comparison with prothrombin time, platelet count and the age-platelet index. Dig Dis Sci.

[B16] Thabut D, Simon M, Myers RP, Messous D, Thibault V, Imbert-Bismut F, Poynard T (2003). Noninvasive prediction of fibrosis in patients with chronic hepatitis C. Hepatology.

[B17] Le Calvez S, Thabut D, Messous D, Munteanu M, Ratziu V, Imbert-Bismut F, Poynard T (2004). The predictive value of Fibrotest vs. APRI for the diagnosis of fibrosis in chronic hepatitis C. Hepatology.

[B18] Halfon P, Imbert-Bismut F, Messous D, Antoniotti G, Benchetrit D, Cart-Lamy P, Delaporte G, Doutheau D, Klump T, Sala M, Thibaud D, Trepo E, Thabut D, Myers RP, Poynard T (2002). A prospective assessment of the inter-laboratory variability of biochemical markers of fibrosis (FibroTest) and activity (ActiTest) in patients with chronic liver disease. Comp Hepatol.

[B19] Rossi E, Adams L, Prins A, Bulsara M, de Boer B, Garas G, MacQuillan G, Speers D, Jeffrey G (2003). Validation of the FibroTest biochemical markers score in assessing liver fibrosis in hepatitis C patients. Clin Chem.

[B20] Imbert-Bismut F, Messous D, Thibaut V, Myers RB, Piton A, Thabut D, Devers L, Hainque B, Mercadier A, Poynard T (2004). Intra-laboratory analytical variability of biochemical markers of fibrosis (Fibrotest) and activity (Actitest) and reference ranges in healthy blood donors. Clin Chem Lab Med.

[B21] Munteanu M, Messous D, Thabut D, Imbert-Bismut F, Jouys M, Massard J, Piton A, Bonyhay L, Ratziu V, Hainque B, Poynard T (2004). Intra-individual fasting versus postprandial variation of biochemical markers of liver fibrosis (Fibrotest) and activity (Actitest). Comp Hepatol.

[B22] The French METAVIR Cooperative Study Group (1994). Intraobserver and interobserver variations in liver biopsy interpretation in patients with chronic hepatitis C. Hepatology.

[B23] Bedossa P, Poynard T (1996). An algorithm for the grading of activity in chronic hepatitis C. The METAVIR Cooperative Study Group. Hepatology.

[B24] Poynard T, Imbert-Bismut F, Ratziu V, Myers RP, Di Martino V, Thabut D, Moussalli J, Benhamou Y (2003). Fibrotest even better than liver biopsy? [Electronic letter. Response]. Clin Chem.

[B25] Poynard T (2003). Cost effectiveness of pegylated interferon alpha 2b and ribavirin combination in chronic hepatitis C [letter]. Gut.

[B26] Callewaert N, Van Vlierberghe H, Van Hecke A, Laroy W, Delanghe J, Contreras R (2004). Noninvasive diagnosis of liver cirrhosis using DNA sequencer-based total serum protein glycomics. Nat Med.

[B27] Poynard T, Imbert-Bismut F, Ratziu V, Naveau S, Thabut D, Lebrec D, Halfon P, Zoulim F, Bourliere M, Messous D, Thibaut V, Muntenau M (2003). An overview of biochemical markers' (Fibrotest-Actitest) diagnostic value in chronic liver diseases: a non-invasive alternative to liver biopsy [abstract]. Hepatology.

[B28] Thabut D, Imbert-Bismut F, Cazals-Athem D, Moreau R, Messous D, Ratziu V, Munteanu M, Valla D, Lebrec D, Poynard T (2003). Diagnostic value of fibrosis biochemical markers (Fibrotest) for the prediction of portal hypertension in liver disease [abstract]. Hepatology.

[B29] Thabut D, Trabut JB, Le Calvez S, Thibaut V, Massard J, d'Arondel C, Moussalli J, Munteanu M, Imbert-Bismut F, Messous D, Benhamou Y, Ratziu V, Poynard T (2003). Diagnostic value of fibrosis biochemical markers (Fibrotest) for the screening of oesophageal varices in patients with chronic liver disease [abstract]. Hepatology.

[B30] Halfon P, Bourliere M, Deydier R, Botta-Fridlund D, Portal I, Renou C, JJ Bertrand JJ, Tran A, A Rosenthal A, Rotily M, A Sattonet A, Ouzan D (2003). Independent prospective multicenter validation of biochemical markers (Fibrotest-Actitest) for the prediction of liver fibrosis and activity in patients with chronic hepatitis C [abstract]. Hepatology.

[B31] Myers RP, Tainturier MH, Ratziu V, Piton A, Thibault V, Imbert-Bismut F, Messous D, Charlotte F, Di Martino V, Benhamou Y, Poynard T (2003). Prediction of liver histological lesions with biochemical markers in patients with chronic hepatitis B. J Hepatol.

[B32] Murawaki Y, Ikuta Y, Okamoto K, Koda M, Kawasaki H (2001). Diagnostic value of serum markers of connective tissue turnover for predicting histological staging and grading in patients with chronic hepatitis C. J Gastroenterol.

[B33] Fortunato G, Castaldo G, Oriani G, Cerini R, Intrieri M, Molinaro E, Gentile I, Borgia G, Piazza M, Salvatore F, Sacchetti L (2001). Multivariate discriminant function based on six biochemical markers in blood can predict the cirrhotic evolution of chronic hepatitis. Clin Chem.

[B34] Poynard T, Bedossa P (1997). Age and platelet count: a simple index for predicting the presence of histological lesions in patients with antibodies to hepatitis C virus. METAVIR and CLINIVIR Cooperative Study Groups. J Viral Hepat.

[B35] Ono E, Shiratori Y, Okudaira T, Imamura M, Teratani T, Kanai F, Kato N, Yoshida H, Shiina S, Omata M (1999). Platelet count reflects stage of chronic hepatitis C. Hepatol Res.

[B36] Hintze JL (2003). NCSS 2003 User Guide Kaysville Utah: Number Cruncher Statistical Systems.

